# Kinetic, kinematic, magnetic resonance and owner evaluation of dogs before and after the amputation of a hind limb

**DOI:** 10.1186/s12917-016-0644-5

**Published:** 2016-01-25

**Authors:** Vladimir Galindo-Zamora, Verena von Babo, Nina Eberle, Daniela Betz, Ingo Nolte, Patrick Wefstaedt

**Affiliations:** Small Animal Hospital, University of Veterinary Medicine Hannover, Foundation, Bünteweg 9, D-30559 Hannover, Germany; Small Animal Clinic, Faculty of Veterinary Medicine, National University of Colombia, Carrera 30 # 45-03 (Ciudad Universitaria), Bogotá, Colombia

**Keywords:** Hind limb amputation, Kinetic and kinematic analyses, Magnetic resonance imaging, Owner evaluation

## Abstract

**Background:**

The amputation of a limb is a surgical procedure that is regularly performed in small animal practice. In spite of several clinical reports indicating high owner satisfaction after limb amputation in dogs, an amputation is still very critically seen by the owners, and even by some veterinarians, due to the lack of accurate information about the recovery of amputee patients. Thus, the objective of this study was to prospectively evaluate, both objectively and subjectively, the recovery outcome of dogs undergoing a hind limb amputation. Twelve patients in which a hind limb amputation was scheduled were studied. Kinetic and kinematic gait analyses were performed before the amputation, and 10, 30, 90 and 120 days after surgery. Magnetic resonance (MR) examination of the contralateral stifle joint was performed before and 120 days after amputation. The subjective impressions of the owners were gathered at the same examination times of the gait analyses.

**Results:**

Kinetic data showed a redistribution of the load to all remaining limbs after the amputation; ten days after the procedure patients had already established their new locomotory pattern. Kinematic data showed significant differences between sessions in the mean angle progression curves of almost all analyzed joints; however, the ranges of motion were very similar before and after the amputation, and remained constant in the subsequent sessions after the amputation. No changes in the signal intensity of the soft tissues evaluated, and no evidence of cartilage damage or osteoarthritis was seen on the MR examination of the contralateral stifle. Owners evaluated the results of the amputation very positively, both during and at the end of the study.

**Conclusions:**

Dogs had a quick adaptation after a hind limb amputation, and the adaptation process began before the amputation was performed. This happened without evidence of morphologic changes in the contralateral stifle joint, and with a very positive evaluation from the owner.

**Electronic supplementary material:**

The online version of this article (doi:10.1186/s12917-016-0644-5) contains supplementary material, which is available to authorized users.

## Background

The amputation of a limb is a surgical procedure that is regularly performed in small animal practice. Severe trauma and limb tumors are the most common reasons for performing an amputation; other indications include chronic osteomyelitis, neurological dysfunctions such as sciatic neuropathy and brachial plexus paralysis, congenital limb deformities, vascular disease and arteriovenous fistulas [1–3].

In spite of several clinical reports indicating high owner satisfaction after limb amputation in dogs [4–7], an amputation is still very critically seen by the owners, and even by some veterinarians. Particularly, owners have the tendency to think that the procedure may affect the animals emotionally, as it indeed happens in people [8], or that it will be disabling for them. Besides, owners are often worried about the possibility of overload of the remaining limbs, leading to hypothetical secondary joint pathologies. Thus, many owners are reluctant to have their dog amputated and reject the amputation as an alternative to euthanasia or take the decision only after the patient has gone through a painful surgical and/or medical treatment process.

The lack of objective information prevents the veterinarian from providing the owners with accurate information about these concerns. A hesitant veterinarian might then play a role in the owner deciding against the amputation. There is only one previously published report objectively evaluating the gait of amputated dogs [9]. In that study, force plate analyses were carried out to measure ground reaction forces (GRF) and contact times in a population of 10 large-breed dogs which had a limb amputation (five forelimbs and five hind limbs). Additionally, the center of gravity was calculated in those patients. It was found that the absence of a limb caused statistically significant changes in the GRF, impulses and contact times of the remaining limbs and the location of the animal’s centre of gravity, in comparison to a control group of 22 healthy dogs. However, there are no prospective studies with animals which are planned to be amputated, and no study has been performed objectively evaluating kinematics (joint movement) or possible joint changes after a hind limb amputation in dogs.

The general objective of the present study was therefore to prospectively characterize the recovery outcome of dogs undergoing a hind limb amputation. In order to evaluate the motion and weight bearing characteristics, as well as the duration of adaptation to the three-legged gait, kinematic and kinetic analyses were carried out. Furthermore, MR images of the remaining contralateral femorotibial (stifle) joint were made before and 4 months after the amputation, in order to investigate possible changes in joint morphology, due to a hypothetic weight bearing overload of this limb.

It was hypothesized that there would be marked changes both in the kinetic and the kinematic parameters after the amputation, but that those changes would not impair the ability of the animal to lead a normal life. Based on our clinical experience and some of the aforementioned studies [4–7], it was also hypothesized that there would not be any changes in the contralateral stifle on the MR examination. Thus, after the initial reluctance to the amputation, owners would be satisfied with the procedure.

## Methods

This study was carried out in accordance with the German Animal Welfare Guidelines and was approved by the Ethics Committee of the Lower Saxony State Office for Consumer Protection and Food Safety (Approval Number: 10A071). All owners agreed to their dogs participation in the study and signed a consent form.

### Patients

All dogs presented to the Small Animal Hospital of the University of Veterinary Medicine Hannover, Foundation (Germany), between March 2010 and October 2011, for a hind limb amputation were included in the study. In total, 12 patients were enrolled. Two additional patients were not included due to aggressiveness in one case, and presence of metallic orthopedic implants in both stifle joints, making it unadvisable to perform the MR examination, in the other case.

Before surgery a thorough physical examination, including an orthopedic and neurologic examination of all remaining limbs and the spine, was performed to rule out any disease which might obscure the results. This examination was repeated 10, 30, 90 and 120 days after the amputation. It was planned that, in case an abnormality was suspected, all necessary diagnostic examination tools would be used to determine the type and location of such an abnormality and its possible relationship with the amputation.

### Surgical procedure

On the amputation day, physical status was determined based on the physical examination, blood work and other diagnostic tests as needed. Based on the American Society of Anesthesiologists (ASA) physical status classification system, all patients were classified as ASA 2 (patients with local or mild systemic disease). The animals were premedicated using a combination of levomethadone (0.6 mg/kg) 1 and diazepam (0.5 mg/kg) 2; anesthesia was induced with propofol dosed to effect (1–4 mg/kg) ^.^^3^ After orotracheal intubation, anesthesia was maintained with isoflurane 4 in a 1:1 oxygen: air mixture adjusted according to the physical signs of anesthetic depth (end-tidal isoflurane 0.7-1.5 vol %) and a continuous rate infusion (CRI) of fentanyl (0.16 μg/kg/min),^5^ lidocaine (50 μg/kg/min) 6 and ketamine (10 μg/kg/min).^7^ Additionally, a preoperative epidural anesthesia with bupivacaine (0.5 mg/kg) 8 and morphine (0.1 mg/kg) 9 and a intraoperative sciatic nerve block with lidocaine (1 mg/kg) 10 were performed. For postoperative analgesia the aforementioned CRI of fentanyl, lidocaine and ketamine was used for 24 h, and carprofen (4 mg/kg)^11^ was initiated the day of the surgery and continued for 10 additional days.

The surgical procedure was performed by disarticulation of the hip, as described elsewhere [2]. The dogs remained in the hospital for approximately 5 days.

### Kinetic and kinematic gait evaluation

Kinetic (forces) and kinematic (movement) gait analysis was performed one to three days before the amputation, as well as 10, 30, 90 and 120 days after surgery.

Kinetics were measured using a specially designed treadmill^12^ consisting of four separate belts, each of them with an integrated force plate underneath. This design allowed the simultaneous measurement of all limb forces.

**Fig. 1:**
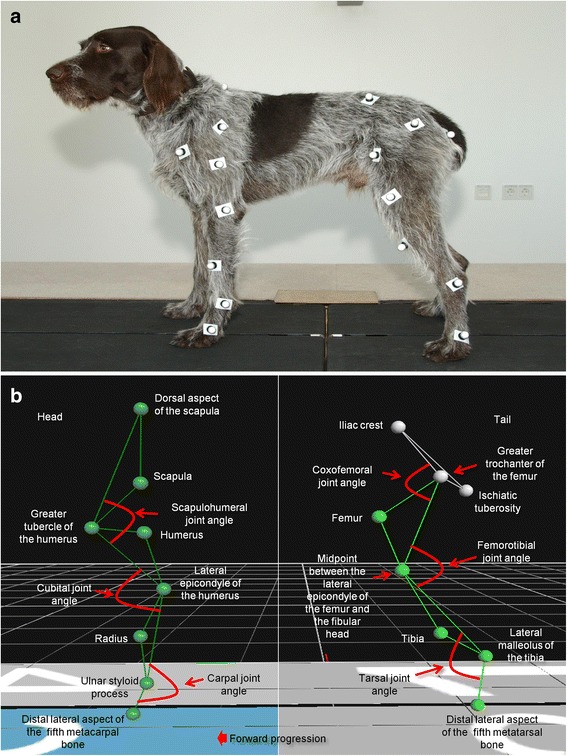
**a** Example of the localization of the retro-reflective markers on a healthy patient; **b** Illustration of the localization of the retro-reflective markers on the anatomical reference points and the measured angles

Kinematic analysis was performed with the aid of retro-reflective markers (Ø 16 mm reflective markers)^13^ positioned on 24 anatomic landmarks (8 per remaining limb), using double-sided adhesive tape; the location of these markers has been previously described [10, 11] and is illustrated in Fig. 1. Six high-speed infrared cameras^14^ were used to record marker movement in all three remaining limbs simultaneously, as the animals were walking at a controlled speed (measurement frequency: 100 Hz). Before each measurement, static and dynamic camera calibration was performed using an L-shaped calibration device.^15^

On each gait analysis session, patients were gently introduced to the gait on the treadmill; on the first day, a speed at which each individual patient walked comfortably on the treadmill was determined; on each subsequent session the patient was evaluated using the same speed, ranging from 0.5 to 0.8 m/s. During each gait analysis session, two to six trials were recorded, each with a duration of approximately 30 s, until at least one valid trial was obtained. A valid trial was defined as 10 consecutive regular steps, in which the dog walked smoothly, without any external forces from the handler being applied, with all paws landing on the appropriate force plate, without overstepping. Video recording was performed, to ensure that the steps were appropriate for analysis.

Both kinetic and kinematic data were simultaneously recorded using commercially available software.^16^

Ten consecutive steps were afterwards analyzed for the following kinetic parameters: peak vertical force (PFz), mean vertical force (MFz), and vertical impulse (integral [IFz]). All forces were normalized to the individual body weight of each dog and data were expressed as percentage of body weight (%BW). Mean ± standard deviation (SD) was calculated from 10 valid consecutive steps. Afterwards, load redistribution (LR) was calculated for each forelimb and remaining hind limb, for each measured parameter (PFz, MFz, IFz), using the following equation (according to Steiss et al. [12]): % load bearing = Fz of the limb/total Fz of all limbs*100. Other kinetic parameters were not calculated to avoid overloading this section with too much information. The kinetic data were processed using commercial software^17^ and exported to a commercially available spreadsheet.^18^

In order to process the kinematic data in Vicon Nexus, all markers were labeled in a trial. Then, 10 valid foot strikes were marked manually to define the gait cycle (stance and swing phases) of each limb. Using a 2-dimensional (2-D) model, projected flexion and extension angles of each remaining joint were calculated: contralateral (with respect to the amputated hind limb) scapulohumeral joint, contralateral cubital joint, contralateral carpal joint, ipsilateral (with respect to the amputated hind limb) scapulohumeral joint, ipsilateral cubital joint, ipsilateral carpal joint, contralateral coxofemoral joint, contralateral femorotibial joint and contralateral tarsal joint. Measured angles are illustrated in Fig. 1. In order to compare the movement pattern of each analyzed joint, the gait cycles were normalized to 100 in all dogs and displayed as percentage of one whole stride. The mean joint angle (±SD) and the range of motion (±SD) of the aforementioned joints were calculated from the mean joint angle progression curves (MJAPC) calculated from the 10 strides per dog. Mean joint angles and range of motion were used since the reader can easily understand their comparison between sessions and to avoid overloading the manuscript with too much data. The kinematic data were processed using commercial software^19^ and then exported to a commercially available spreadsheet^18^.

### MR evaluation of the contralateral stifle joint

The MR examination was performed under general anesthesia before and 120 days after amputation. The anesthetic protocol was the same described above, excluding local anesthetics and CRIs. The animals were positioned in lateral recumbency with the limb to be examined in a non-dependent position and the stifle joint at an angle of ~135°. Using a state-of-the-art 3 T MR scan,^20^ images were obtained from the contralateral stifle. Small (11 cm Ø) surface ring coils (Achieva 3.0 T Musculoskeletal SENSE Flex S coil 2 elements) were used as image enhancers; these were positioned parallel to each other, lateral and medial to the examined stifle, with the joint centered between the two coils. The MR protocol used included a 3-D (3-dimensional) PDW (proton-density weighted) acquisition sequence, which was afterwards reconstructed in sagittal, dorsal (parallel to patella ligament) and transversal (parallel to tibial plateau) planes (slices every 2 mm), a PDW HR (high-resolution) TSE (turbo spin echo) SENSE (sensitivity encoding for fast MR) sequence in sagittal plane (slices every 2 mm),, a PDW HR SPAIR (spectrally adiabatic inversion recovery) SENSE in sagittal plane (slices every 2 mm), and a T1-weighted TSE clear (constant level appearance) sequence in sagittal plane (slices every 1.8 mm).

This protocol had been previously standardized and regarded as suitable for use in clinical cases, since diagnostic image quality is optimal and acquisition time is only 22 min (total examination time is about 40 min including positioning, reference scan, survey, and sequence planning).

Using a high-resolution diagnostic screen^21^ the images were assessed by a trained evaluator (VGZ), who looked for changes in the signal intensity of the cranial cruciate ligament (CrCL), the caudal cruciate ligament (CdCL) and the lateral and medial menisci. Possible changes in the cartilage surfaces, as well as evidence of osteoarthritic changes were also evaluated in the lateral and medial femoral condyles, femoral trochlear groove, patella and tibial plateau.

It was expected that, due to a possible underlying metastatic disease, some patients could die or be euthanized before the end of the study; if that was the case, it was planned to ask the owner to authorize the MR examination postmortem.

### Owner evaluation of patient comfort

The owner was requested to fill out an evaluation form (modified from Hielm-Björkman et al. [13]) before the amputation and 10, 30, 90 and 120 days after the procedure, in order to gather his/her (subjective) impressions with regard to patient comfort and recovery. At the end of the study (day 120), owners filled out a questionnaire to assess their final impression regarding the degree of activity and life quality of the dog, and their general impression of and satisfaction with the procedure; besides, owners were encouraged to make further comments. It was planned that if the animal died before the end of the study, an appropriate moment would be looked for to ask the owner to fill out the questionnaire. The questions of the questionnaire were adapted from Carberry and Harvey [4], Withrow and Hirsch [5], von Werthern et al. [6] and Kirpensteijn et al. [7]. The owners’ assessment of patient comfort and the final questionnaire were made in German and translated into English as accurately as possible.

### Statistical methods

Due to the small sample size and very heterogeneous patient population included in this study, it was decided to use non-parametric statistics. Thus, data were analyzed using a Kruskal-Wallis one-way ANOVA test to compare between sessions; when statistically significant differences were found, a Wilcoxon signed-rank test for paired observations was performed to determine which session was different. All tests were considered statistically significant if *p* < 0.05 and were performed using standard statistical software.^22^ Descriptive statistics were calculated using a commercially available spreadsheet^18^, where appropriate.

## Results

### Clinical data

Breed, sex, age, reason to amputate and performed evaluations of the 12 patients enrolled in this study are illustrated in Table 1. As can be seen in this table, the most common reason for performing the amputation was a tumor, followed by trauma and one surgical complication. Six right and six left hind limbs were amputated. Nine patients survived until the end of the study. Due to the underlying metastatic disease, one animal (patient 5) was euthanized 36 days after the amputation and another one (patient 6) died 120 days after the procedure. One dog (patient 8) died unexpectedly 22 days after the amputation due to abdominal bleeding caused by a previously asymptomatic and undiagnosed hepatic hemangiosarcoma.

**Table 1:** Patients included in this study

Patient	Breed	Sex	Age	Weight	Reason to amputate	Gait analyses					PO MR
			(Years)	(kg)		Pre	10	30	90	120	
1	Boxer	Male	8	32	Osteosarcoma	+	+	+	+	+	+
2	Labrador	Female	3	31	Rhabdomyosarcoma	+	+	+	+	+	+
3	Mixed-breed dog	Female	4	32	Osteosarcoma	+	+	+	+	+	+
4	Mixed-breed dog	Male	1	20	Severe soft tissue trauma	+	+	+	+	+	+
5	Mixed-breed dog	Male	12	31	Osteosarcoma	+	+	+	E	-	-
6	Swiss Mountain dog	Female	10	39	Osteosarcoma	+	+	+	+	E	-
7	Bernese Mountain dog	Male	2	40	Femoral fracture nonunion	-	-	-	-	-	+
8	German Shepherd mix	Male	7	26	Severe soft tissue trauma	+	+	E	-	-	-
9	Mixed-breed dog	Female	8	13	Osteosarcoma	+	+	+	+	+	+
10	Mixed-breed dog	Female	11	8	Malignant sarcoma	+	+	+	+	+	+
11	Landseer	Female	2	54	Fibrosarcoma	+	+	+	+	+	-
12	Mixed-breed dog	Female	8	49	Osteosarcoma	+	+	+	+	+	+

PO MR: Postoperative magnetic resonance scan; +: Performed; −: Not performed; E: Euthanasia

Patient 12 presented bilateral hip osteoarthritis; however, it was asymptomatic, and no signs of pain, lameness or difficulty to stand up were detected before or after the amputation. All other patients showed no abnormalities in the physical examination of the remaining limbs. No patient showed abnormalities on the physical examination of the spine throughout the study.

### Kinetic and kinematic gait evaluation

**Fig. 2:**
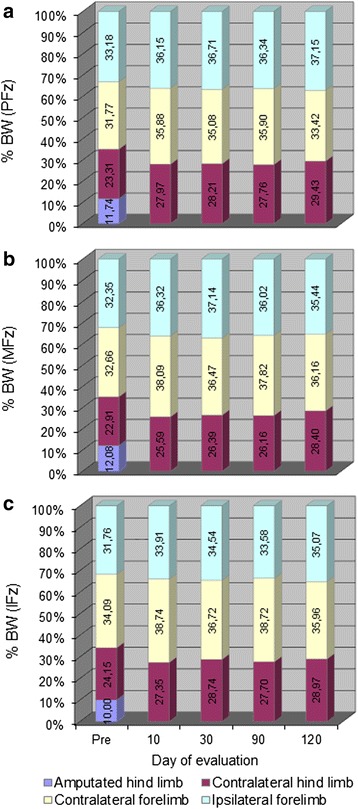
Load redistribution (LR) averages for the **a** peak (PFz); **b** mean (MFz); and **c** integral (IFz) forces. The values in the bars indicate the mean % body weight (BW) loaded by each limb for each calculated parameter

**Fig. 3:**
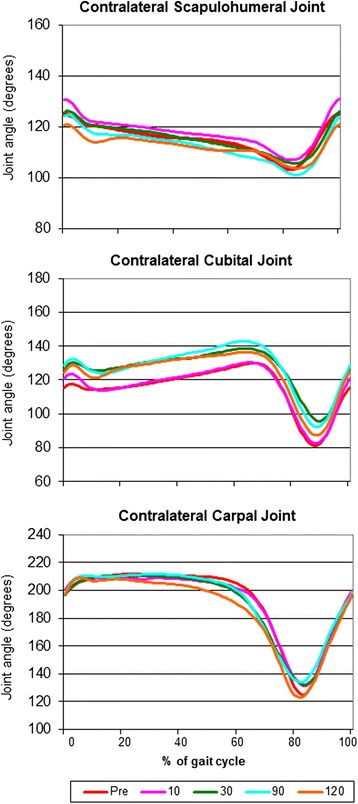
Mean joint angle progression curves of the contralateral (with respect to the amputated hind limb) scapulohumeral joint, cubital joint and carpal joint. Note the similarity of the curves before and after amputation

**Fig. 4:**
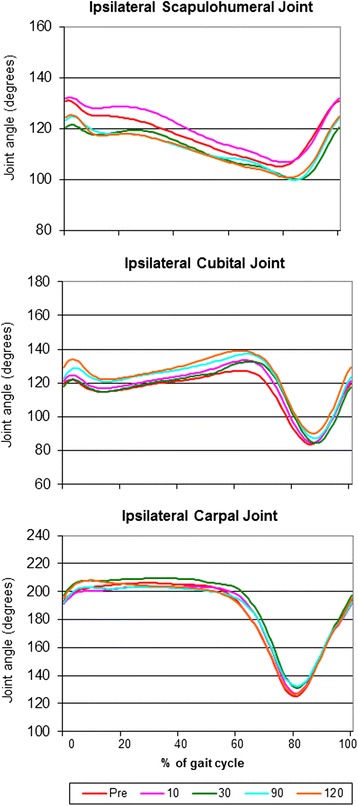
Mean joint angle progression curves of the ipsilateral (with respect to the amputated hind limb) scapulohumeral joint, cubital joint and carpal joint. Note the similarity of the curves before and after amputation

**Fig. 5:**
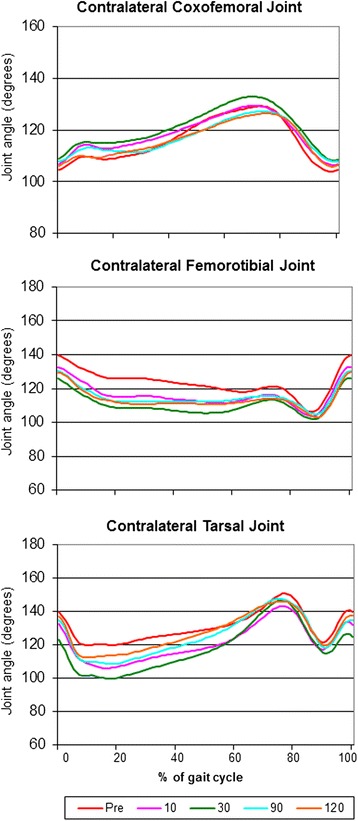
Mean joint angle progression curves of the contralateral (with respect to the amputated hind limb) coxofemoral joint, femorotibial joint and tarsal joint. Note the similarity of the curves before and after amputation

The results of the kinetic and kinematic evaluations are presented in Figs. 2, 3, 4 and 5 and Tables 3 and 4. It should be noted that, although nine patients survived until the last examination day, the kinetic and kinematic data were not available from all of them. One animal (Patient 7) refused to walk on the treadmill and others (e.g. Patient 4) walked intermittently in such a way that some trials were not valid for analysis; even though these animals could walk and run perfectly fine on solid ground, they were afraid of walking on the treadmill, apparently due to the movement of the belts. Moreover, although all owners were extremely cooperative, when some of them had the impression that their dog was afraid or tired, they were reluctant to allow their pets to be walked on the treadmill long enough to record valid trials. The number of patients evaluated on each session is also indicated in Tables 3 and 4.

Kinetic data showed that 10 days after amputation there was redistribution of the load to all remaining limbs (Fig. 2 and Table 2). The values and pattern of load shifting are represented in Fig. 2. The recorded PFz, MFz and IFz values showed no remarkable changes during the remaining examination time points, indicating that 10 days after the amputation the patient had already reached its new locomotory pattern. This was true for all patients including the lightest (8 kg) and the heaviest (54 kg) ones. There were no statistically-significant differences between sessions (Table 2).

**Table 2:** Results of the kinetic analysis

		Pre	10	30	90	120	*p*
		(*n* = 11)	(*n* = 11)	(*n* = 10)	(*n* = 9)	(*n* = 8)	
PFz contralateral forelimb							
	Mean	64.88	71.45	67.69	69.78	66.74	0.252
	SD	7.00	4.73	8.71	6.44	8.27	
PFz ipsilateral forelimb							
	Mean	67.78	71.97	70.86	70.64	74.18	0.796
	SD	9.03	5.90	9.29	5.60	11.56	
PFz contralateral hind limb							
	Mean	47.60	55.68	54.44	53.96	58.75	0.454
	SD	11.71	10.07	9.62	13.69	15.36	
MFz contralateral forelimb							
	Mean	47.55	52.71	49.33	50.70	48.49	0.327
	SD	7.13	4.71	6.84	4.37	7.45	
MFz ipsilateral forelimb							
	Mean	47.10	50.27	50.25	48.30	47.53	0.816
	SD	6.93	5.30	7.79	3.43	9.55	
MFz contralateral hind limb							
	Mean	33.37	35.41	35.70	35.08	38.09	0.865
	SD	7.02	6.27	6.42	10.38	10.48	
IFz contralateral forelimb							
	Mean	30.28	32.09	30.41	30.07	28.82	0.945
	SD	7.28	7.46	9.10	8.45	7.54	
IFz ipsilateral forelimb							
	Mean	28.20	28.09	28.61	26.08	28.10	0.967
	SD	5.90	7.12	6.57	6.31	6.81	
IFz contralateral hind limb							
	Mean	21.45	22.65	23.81	21.51	23.21	0.999
	SD	7.28	7.00	8.19	5.23	7.62	

*p*: *p* value of the Kruskal-Wallis test; PFz: Peak vertical force; MFz: Mean vertical force; IFz: vertical impulse (integral); SD: Standard deviation

With regard to the kinematic gait analysis, even though the patients walked smoothly on the treadmill (Additional file 1: Video 1), there were significant differences between sessions in the means of almost all joint angles (Table 3). It is important to note that there were also marked variations within a patient in the same session (not shown in Table 3). The MJAPC showed a similar pattern between sessions (Figs. 3, 4 and 5), but these showed marked individual variations (not shown).

**Table 3:** Results of the kinematic analysis

		Pre (*n* = 11)	10 (*n* = 11)	30 (*n* = 10)	90 (*n* = 9)	120 (*n* = 8)	*p*
Contralateral scapulohumeral joint	Mean ± SD	115.2 ± 5.67	117.9 ± 5.83	115.4 ± 5.54	112.7 ± 6.09	112.3 ± 4.24	<0.001
	ROM ± SD	27.68 ± 7.48	31.46 ± 8.12	29.51 ± 6.73	31.49 ± 6.26	26.07 ± 6.04	0.478
Contralateral cubital joint	Mean ± SD	114.9 ± 12.93	115.9 ± 12.82	126.5 ± 11.59	127.2 ± 13.67	123.6 ± 13.91	<0.001
	ROM ± SD	57.98 ± 15.56	61.8 ± 11.64	56.51 ± 14.74	60.75 ± 13.06	62.74 ± 9.60	0.898
Contralateral carpal joint	Mean ± SD	192.2 ± 26.85	191.5 ± 24.89	190.1 ± 26.03	191.5 ± 25.66	185.8 ± 26.88	<0.001
	ROM ± SD	99.38 ± 19.62	97.45 ± 11.28	104.7 ± 10.83	94.25 ± 13.65	101.1 ± 8.17	0.666
Ipsilateral scapulohumeral joint	Mean ± SD	117.5 ± 7.92	120.1 ± 8.33	111.9 ± 6.87	112.4 ± 6.94	112.1 ± 7.12	<0.001
	ROM ± SD	34.11 ± 5.69	32.2 ± 4.35	29.77 ± 4.80	32.17 ± 2.71	31.06 ± 5.03	0.654
Ipsilateral cubital joint	Mean ± SD	114.6 ± 12.28	117.6 ± 13.33	116.7 ± 12.94	121.5 ± 13.63	123.8 ± 13.58	<0.001
	ROM ± SD	55 ± 11.81	57.96 ± 11.92	57.23 ± 12.69	59.84 ± 12.73	57.78 ± 11.82	0.916
Ipsilateral carpal joint	Mean ± SD	185.5 ± 26.36	185.6 ± 24.54	190.6 ± 25.54	185.9 ± 23.23	185.5 ± 26.05	<0.001
	ROM ± SD	92.65 ± 19.14	91.83 ± 18.76	91.01 ± 15.71	82.42 ± 12.51	93.36 ± 10.05	0.630
Contralateral coxofemoral joint	Mean ± SD	116.1 ± 8.37	118.4 ± 6.96	120.8 ± 7.516	116.8 ± 6.29	116.1 ± 6.64	<0.001
	ROM ± SD	30.25 ± 6.22	27.47 ± 9.90	30.08 ± 9.71	25.17 ± 9.09	24.89 ± 6.27	0.501
Contralateral femorotibial joint	Mean ± SD	123.2 ± 7.48	115.8 ± 6.62	110.4 ± 5.76	114.7 ± 5.55	113.3 ± 5.68	<0.001
	ROM ± SD	42.4 ± 4.28	37.67 ± 8.44	37.82 ± 7.34	40.8 ± 12.16	34.83 ± 8.31	0.299
Contralateral tarsal joint	Mean ± SD	130.5 ± 8.98	121 ± 10.93	117.8 ± 14.07	124.9 ± 12.21	127.2 ± 10.75	<0.001
	ROM ± SD	47.74 ± 10.2	50.57 ± 10.52	61.27 ± 14.25	57.73 ± 11.24	48.04 ± 14.17	0.143

Mean: mean joint angle calculated from the mean joint angle progression curves; SD: Standard deviation; ROM = Range of Motion; *p* = *p* value of the Kruskal-Wallis test

Despite all these different kinematic results, ROMs of all analyzed joints were very similar before and after amputation and remained constant in the subsequent sessions after the amputation, without significant differences between sessions (Table 3).

### MR evaluation of the contralateral stifle joint

**Fig. 6:**
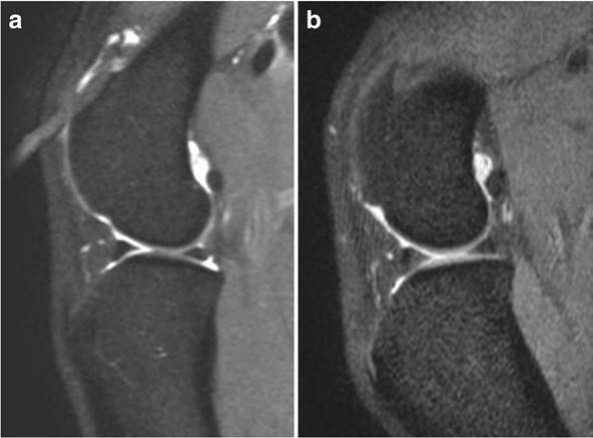
Example of the magnetic resonance examination of the stifle in one patient before (**a**) and 120 days after the amputation (**b**). No changes could be detected in the joint 120 days after the procedure

Postoperative MR examination was possible in eight patients. Although nine patients survived until the end of the study, severe metastatic disease was detected in patient 11 on day 120, and the MR examination was not performed. Postmortem MR examination was not possible in any case, as the owners were very sensitive about their pet’s death, and they elected to dispose the patients’ dead bodies themselves. No changes in the signal intensity of the CrCL, CdCL or the lateral and medial menisci were found, in comparison with the preoperative MR images. No changes in the cartilage surface and no evidence of osteoarthritic changes were found (Fig. 6).

### Owner evaluation of patient comfort

The results of the owners’ assessment of patient comfort are presented in Table 4 and show a clear tendency of the patients to improve after amputation. Patient movement after amputation is exemplified with Additional file 2: Video 2. Ten owners answered the final questionnaire (Table 5). This questionnaire revealed a high degree of owner satisfaction with the amputation result.

**Table 4:** Owner assessment of patient comfort^a^ (first part)

	Pre (*n* = 12)	10 (*n* = 12)	30 (*n* = 11)	90 (*n* = 10)	120 (*n* = 10)
Attitude					
Very bright (score 0)	4	2	6	4	3
Alert (score 1)	5	8	5	6	7
Neither alert nor indifferent (score 2)	3	2	0	0	0
Indifferent (score 3)	0	0	0	0	0
Depressed (score 4)	0	0	0	0	0
*Cumulative score for attitude*	*11*	*12*	*5*	*6*	*7*
Willingness to move (general)					
Very willing (score 0)	2	3	6	5	5
Willing (score 1)	4	8	5	4	5
Hesitant (score 2)	4	1	0	1	0
Reluctant (score 3)	1	0	0	0	0
Does not move (score 4)	1	0	0	0	0
*Cumulative score for willingness to move (general)*	*19*	*10*	*5*	*6*	*5*
The dog …					
… lies down …					
Easily (score 0)	3	4	7	9	9
Carefully (score 1)	5	7	4	1	1
Slowly (score 2)	1	1	0	0	0
with difficulty (score 3)	2	0	0	0	0
with a lot of difficulty (score 4)	1	0	0	0	0
*Cumulative score for the dog lies down…*	*17*	*7*	*4*	*1*	*1*
… stands up …					
Easily (score 0)	4	2	4	5	6
Carefully (score 1)	3	10	6	4	2
Slowly (score 2)	2	0	1	1	2
with difficulty (score 3)	2	0	0	0	0
with a lot of difficulty (score 4)	1	0	0	0	0
*Cumulative score for the dog stands up…*	*17*	*10*	*8*	*6*	*6*
Willingness to move after resting					
Very willing (score 0)	0	4	4	4	4
Willing (score 1)	5	7	6	5	5
Hesitant (score 2)	6	0	1	1	1
Reluctant (score 3)	1	1	0	0	0
Does not move (score 4)	0	0	0	0	0
*Cumulative Score for willingness to move after resting*	*20*	*10*	*8*	*7*	*7*
Willingness to move after exercising					
Very willing (score 0)	0	0	1	3	2
Willing (score 1)	3	9	6	4	6
Hesitant (score 2)	4	2	3	3	2
Reluctant (score 3)	3	1	1	0	0
Does not move (score 4)	2	0	0	0	0
*Cumulative score for willingness to move after exercising*	*28*	*16*	*15*	*10*	*10*
*Mean score (sum of cumulative scores / patients evaluated)*	*9.3*	*5.4*	*4.1*	*3.6*	*3.6*

^a^Modified from Hielm-Björkman et al. [13]

**Table 5:**
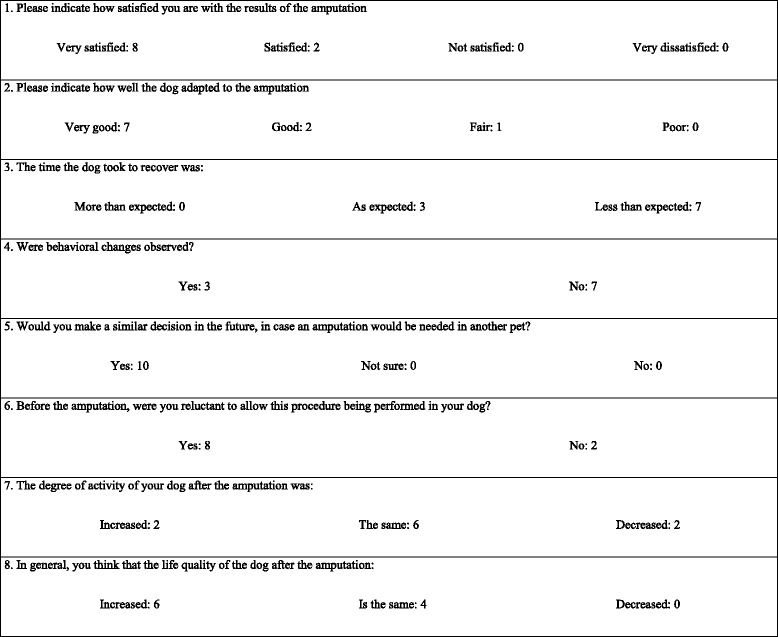
Final owner questionnaire^a^ (*n* = 10)

^a^Adapted from Carberry and Harvey [4], Withrow and Hirsch [5], von Werthern et al. [6] and Kirpensteijn et al. [7]

## Discussion

The main goal of this study was to provide owners and veterinarians with accurate, objective information, not existing previously, about the outcome of the amputated patient (i.e. how the dog adapted to the new locomotory status).

Examination times were chosen using the data of a previous study [7] which indicated that most dogs adapted to the amputation within a month, some within a week, and all within 3 months after surgery. With the last follow-up 120 days after the procedure, a final attempt was made to find any evidence of further gait changes. Since there is the belief that orthopedic disease might occur in the remaining limb after amputation, as a result of a theoretical overload, the contralateral stifle was morphologically assessed using MR images, to evaluate this likelihood. The femorotibial (stifle) joint was chosen, instead of the coxofemoral or tarsal joint, as the former has been extensively investigated, and there is a whole body of literature exemplifying normal and pathologic MR images to compare [14–19].

As expected, a very heterogeneous population was found in this study: different breeds, ages and weights. The main reason for amputating a patient was the presence of a tumor, which agrees with previous studies [6, 7]. The mortality seen in this study was not related the amputation itself.

The kinetic and kinematic analyses revealed that the patients had begun adapting to the new locomotory situation even before the amputation was performed, and that 10 days after the procedure, all major changes had already taken place. It was unfortunate that it was not possible to perform all examinations on all patients. However, exclusively using “good” trials allowed us to obtain reliable results. It should be noted that, even though a valid trial implied that the handler did not exert any external forces, it was not possible to have the animals completely free on the treadmill (see Additional file 1: Video 1). Unfortunately, the willingness to walk or trot or overall activity of the patient were not evaluated in the present study; these factors would have brought additional information about the outcome. It is remarkable that the largest patient (patient 11–54 kg) and the smallest one (patient 10 – 8 kg) adapted equally well. These results agree with the study of Kirpensteijn et al. [7], indicating that, even though subjective, the observations made by the owners of such study were actually very precise. The present study has the advantage of looking at the patients objectively and prospectively, leaving no doubt about the fast adaptation of all animals. The fact that there were no significant changes in the load redistribution after amputation was initially surprising; however, it is easily explainable: all patients were severely lame before the amputation, meaning that the adaptation and compensation to the lack of a hind limb had begun to take place before the amputation was performed. This was even clearer when looking only at those patients which did not load the affected limb at all before surgery. It should also be kept in mind, that the lack of statistically significant changes could have resulted from the low number of dogs included in this study, and therefore in the lack of power of the tests.

The kinematic results (including the statistical analysis) should be interpreted with caution. The fact that significant differences were found in the absolute values of the different joint angles could be explained by the normal variation inherent to motion analysis: marker localization changes lead to changes in joint angles measured. Even though every effort was made to place the markers in the right position, small variations might have occurred, leading to different measurements. However, also huge variations were seen within a session (not shown in Table 3 or Figs. 3, 4 and 5), possibly indicating that the patients adapted to every step they made and in a very irregular manner. Affected balance of the dog during movement and the resulting instability might cause these variations. Another possible explanation is the fact that several gait patterns are possible at a given speed and by a permanent adjustment of the speed of the animal to the treadmill speed [20]. It should also be taken into account that slow speeds were used before the amputation, in order to avoid worsening the pain that patients were already experiencing. After the amputation the animals could have walked on the treadmill more comfortably at faster speeds (personal observations). Even though this might have caused an irregular walking pattern after the procedure, speeds were kept constant to avoid adding a variance factor when measuring the GRF [21]. Finally, the angles measured here can be used to illustrate the movement patterns for the patients in this study, and they cannot be extrapolated to other patient populations. However, our study focuses on determining whether there are variations in the different kinematic parameters before and after amputation in this particular patient population, and that does not seem to be the case.

The similarity in the ROMs before and after surgery is a remarkable finding. The measurement of ROMs seems to be less susceptible to the sources of error commonly found in kinematic studies (misplacement of markers and skin movement), at least in the hind limb [22]. Therefore, the results of the ROMs are more accurate than the absolute measurement of joint angles. That being said, the lack of significant differences between sessions suggests that the patient had also begun to adapt to the new movement situation before amputation, and that this remained stable after the procedure. However, it should also be taken into consideration that joint angular curves in each measurement points are very similar in shape, resulting in stable ROMs; this could also explain why the ROMs showed much less variation than mean joint angles.

The lack of changes in the MR examination of the remaining contralateral stifle after amputation could indicate that an overload in the remaining contralateral hind limb, leading to joint pathology, is not very likely. It was decided to investigate this point, as it is commonly believed by the general public, and even by some veterinarians, that amputating a dog might predispose it to orthopedic abnormalities; the results of our MR (and also our physical examinations) proved otherwise, at least for the stifle. Although 4 months after amputation might possibly be a short time to evaluate joint changes, a previous study describing the experimentally induced rupture of the CrCL in 5 crossbred dogs showed that it is possible to see changes in the cartilage and subchondral bone as early as 4 weeks after the rupture [17]. It is of course difficult to extrapolate such findings to this study, but it could indicate that, if there were ongoing changes in joint morphology, they would be visible 4 months after amputation. Besides, most of our research subjects were oncologic patients, with a (likely) short life span. Measuring cartilage thickness would have been a more accurate method to evaluate subtle joint changes [17]. Unfortunately, this could not be done due to software limitations. In any case, we did not expect to see any changes, as our clinical experience indicates that no changes are seen in the contralateral limb after a hindlimb amputation; nevertheless, it is not known what kind of changes an amputation would cause in patients suffering from degenerative joint disease in the remaining limbs.

With regard to the owner evaluation assessment of the patient after the procedure, the results were as expected: in Table 4, the positive outcome of most patients can be clearly seen. The improvement is especially remarkable in the ability of the dog to lie down and stand up. The lack of improvement or worsening of some parameters for some patients seemed to be related more to the declining general condition of the patient, than to the effects of the amputation itself. The use of an objective tool to measure patient activity, such as an accelerometer, could have provided more accurate information about the outcome and they have been previously used in animals [23]; however, this tool was not available to us.

The responses to the final questionnaire were also as expected: most owners were initially reluctant to have their dogs amputated, but were satisfied with the overall result and the quality of life was considered good; this is in agreement with other studies [4–7]. In the present study, some owners even considered that their pet’s quality of life improved after the amputation, and this might have been related to the removal of the source of pain. The behavioral changes reported by the owners in this study were more small disabilities than behavioral problems. The behavioral problems previously reported [7], such as aggression and anxiety, were not seen in the patients of the present study.

As in previous studies [4, 7], all owners responded that they would have another pet amputated, and none regretted the decision. We believe that an evaluation made by owners whose pets died shortly after the procedure would have been negatively biased and they were not gathered.

The favorable responses of most owners can be explained by the fact that the dogs adapted very soon to their new locomotory situation (kinetic and kinematic analyses) and to the lack of morphologic changes in other joints (as it might be inferred from the lack of morphologic changes in the stifle).

### Limitations

Our study provides new information; however, there were important limitations in this study: the lack of a homogeneous population prevented us from comparing the kinetic and kinematic data with other studies looking at normal patient populations or using a control group. However, it is virtually impossible to perform such a clinical study using a homogeneous population. Additionally, kinematic data are breed-specific [24], and not all breeds have been studied yet. In any case, in the present study there were a high number of mixed-breed dogs, which are very difficult to characterize. The small sample size is another important limitation, which also possibly led to lack of statistical power. The effect of missing measurements for some patients might have also obscured the results. Finally, even though the evaluator was experienced in reading MR images of canine stifles, the lack of a board-certified radiologist for interpretation of the images may have also been a limitation of this study.

## Conclusions

In spite of the limitations, this study provides objective evidence indicating that dogs have a quick adaptation process after a hind limb amputation. The adaptative processes to the new locomotion begin even before the amputation is performed. Since the veterinarian is responsible for providing accurate information before an amputation [2], we strongly believe that this study provides useful information, that will allow veterinarians the possibility to give dog owners more realistic expectations of a hind limb amputation.

## Additional files

Additional file 1:
**Video 1.** Thirty-two kilogram patient walking on the treadmill at 0.8 m/s. Although the walking pattern is very similar between sessions, evident differences can be observed. (MPG 9336 kb) Additional file 2:
**Video 2.** Forty-kilogram dog (patient 7) walking and running 4 months after the amputation. Note that the dog has no difficulty in its movements whatsoever, neither when walking nor when running. (MPG 9560 kb)

## Footnotes

1 L-Polamivet®: Intervet Deutschland GmbH, Unterschleißheim, Germany

2 Diazepam-ratiopharm®: Ratiopharm GmbH, Ulm, Germany

3 Narcofol® 10 mg/mL: CP-Pharma Handelsgesellschaft GmbH, Burgdorf, Germany

4 Isofluran CP®: CP-Pharma Handelsgesellschaft GmbH, Burgdorf, Germany

5 Fentanyl-Janssen® 0.05 mg/mL: Janssen-Cilag GmbH, Neuss, Germany

6 Xylocain® 2 %: AstraZeneca GmbH, Wedel, Germany

7 Ketamin 10 %: Selectavet Dr. Otto Fischer GmbH, Weyarn-Holzolling, Germany

8 Bupivacain-RPR-actavis® 0.5 %: Actavis Deutschland GmbH & Co. KG, Langenfeld, Germany

9 Morphin Hexal® 10 mg/mL: Hexal AG, Holzkirchen, Germany

10 Xylocain® 2 %, AstraZeneca GmbH, Wedel, Germany

11 Rimadyl® Injektionslösung: Pfizer GmbH, Berlin, Germany

12 Treadmill model 4060–80: Bertec Corporation, Columbus, OH, USA

13 Vicon Motion Systems Ltd., Oxford, UK

14 MX3+ camera system: Vicon Motion Systems Ltd., Oxford, UK

15 Vicon Calibration Device: Vicon Motion Systems Ltd., Oxford, UK

16 Vicon Nexus: Vicon Motion Systems Ltd., Oxford, UK

17 MyoResearch XP Master Edition, Noraxon U.S.A. Inc., Scottsdale, AZ, USA

18 Microsoft® Excel 2007

19 Vicon Nexus and Bodybuilder: Vicon Motion Systems Ltd., Oxford, UK

20 Philips Achieva 3.0 T X-series MRI: Philips Healthcare, Hamburg, Germany

21 EIZO RadiForce™ RX211 Medical color LCD monitor: Enzo Nanao Corporation, Hakusan, Ishikawa, Japan

22 GraphPad Prism® Version 4: GraphPad Software, Inc. La Jolla, California, USA
